# Gastrointestinal stromal tumor with secondary thrombocytosis: a case report of a high-risk patient

**DOI:** 10.3389/fmed.2025.1605819

**Published:** 2025-08-05

**Authors:** Na Li, Penghui Liu, Jiwu Guo, Jizhen Wang, Xinling Han, Jie Mao

**Affiliations:** ^1^Lanzhou University Second Clinical Medical College, Lanzhou, China; ^2^Lanzhou University Second Hospital, Lanzhou, China

**Keywords:** gastrointestinal stromal tumor, secondary thrombocythemia, c-KIT, Exon 13, case report

## Abstract

Gastrointestinal stromal tumor (GIST) with secondary thrombocytosis is a rare clinical case, exhibiting specificity in clinical diagnosis and treatment. We report a case of GIST with secondary thrombocythemia to raise clinicians’ attention to this disease. On October 11, 2024, a 58-year-old male patient was admitted to the hospital due to “intermittent right lower abdominal pain with increased bowel movements for more than 1 month.” The patient had no prior history of tumors, chronic inflammatory diseases, hematologic disorders or family history of genetic disorders. MRI-enhanced scans of the small intestine highly indicated a lymphoma of intestinal origin. Small bowel endoscopy and pathological biopsy revealed mild chronic inflammation of the intestinal mucosa, with intact villous architecture, no plasmacytosis, granulomas, or vasculitis, and no indication of GIST. Laboratory tests showed platelet count of 909 × 10^9^/L, white blood cell count of 11.86 × 10^9^/L, neutrophil ratio of 75.10%, lymphocyte ratio of 15.30% and hemoglobin 101 g/L. Bone marrow biopsy microscopically showed a normal number of megakaryocytes without abnormal aggregation and no myelofibrosis, suggesting there was no obvious hematologic malignancy and the thrombocytosis may have been secondary. The patient underwent partial resection of the small intestine and resection of mesenteric lesions on October 18, 2024. The intraoperative frozen section suggested a stromal tumor. The postoperative pathological biopsy suggested a GIST and genetic testing showed a mutation in the c-KIT gene (Exon 13). Postoperatively, the patient was treated with oral imatinib mesylate (400 mg/d) as adjunctive therapy. Three months after surgery, imaging showed no recurrence, platelet decreased and returned to normal levels.

## Highlights

- This case represents a rare instance of gastrointestinal stromal tumor (GIST) harboring a KIT Exon 13 mutation accompanied by secondary thrombocytosis.
- The patient’s platelet levels normalized within 3 months after tumor resection and adjuvant therapy, suggesting a tumor-driven mechanism.
- The patient recovered well, resumed daily activities, and reported satisfactory quality of life without adverse events.
- This may be the first documented Exon 13-mutant GIST case with thrombocytosis, highlighting the need for further molecular subtype-based investigations.

## Introduction

Gastrointestinal stromal tumor (GIST) is a rare gastrointestinal tumor derived from Cajal mesenchymal cells that accounts for approximately 1–2% of all gastrointestinal tumors (1). The development of GIST is mainly associated with mutations in the c-KIT or PDGFRA genes, with Exon 11 mutations in the c-KIT gene being the most common, while Exon 13 mutations are rarer, accounting for only 1–2% of cases (2, 3). The clinical presentation of GIST lacks specificity, patients may present with symptoms such as abdominal pain, gastrointestinal bleeding or obstruction without significant systemic hematologic abnormalities (4). The thrombocythemia is a common hematologic disorder that is divided into two categories: primary and secondary (5). Primary thrombocythemia is usually associated with conditions such as true erythrocytosis and myeloproliferative neoplasms, whereas secondary thrombocytosis is caused by factors such as infection, inflammation, iron deficiency, and malignancies (6–8). In patients with solid tumors, thrombocytosis is often thought to be associated with mechanisms such as tumor-associated inflammatory states, cytokine release and compensatory bone marrow proliferation (9). However, the combination of significant thrombocytosis in patients with GIST is extremely rare, and no case reports of GIST combined with secondary thrombocytosis have been identified in the PubMed database. This article summarizes and reports the treatment process and follow-up information of a patient with small intestinal GIST and secondary thrombocytosis, not only filling the research gap but also providing additional insights into the diagnosis, treatment and study of this disease.

## Case presentation

A 58-year-old male was admitted to the hospital on October 11, 2024 with “intermittent right lower abdominal pain with increased bowel movements for more than 1 month. The patient described a progressive worsening of symptoms, especially abdominal discomfort after activity. In order to seek further diagnosis and treatment, the patient was admitted to the Second Hospital of Lanzhou University. The patient was a manual laborer residing in a rural area. He denied any history of tobacco use, alcohol consumption, or recreational drug use. He reported no significant psychosocial stressors, and his daily functioning prior to the onset of abdominal symptoms was unremarkable. There was no known family history of gastrointestinal tumors, hematologic disorders, or hereditary syndromes. Additionally, he had no recent history of significant weight loss, appetite reduction, or melena, and no previous history of neoplasia, chronic inflammatory diseases, hematologic disorders, infectious diseases, or other familial hereditary conditions.

## Examination

### Physical examination

The patient’s vital signs were stable, the abdomen was flat without deformity, there was no obvious mass on palpation, mild pressure pain in the right lower abdomen without rebound pain, and bowel sounds were normal on auscultation.

### Laboratory examination

Blood count: platelet count of 909 × 10^9^/L (reference range: 100–300 × 10^9^/L), white blood cell count of 11.86 × 10^9^/L, red blood cell count of 4.88 × 10^12^/L, neutrophil ratio 75.10%, lymphocyte ratio 15.30% and hemoglobin 101 g/L.

Coagulation function: mild elevation in D-dimer levels, indicating potential hypercoagulable state.

Inflammatory markers: mildly elevated C-reactive protein and erythrocyte sedimentation rate.

### Imaging examination

**Figure 1:**
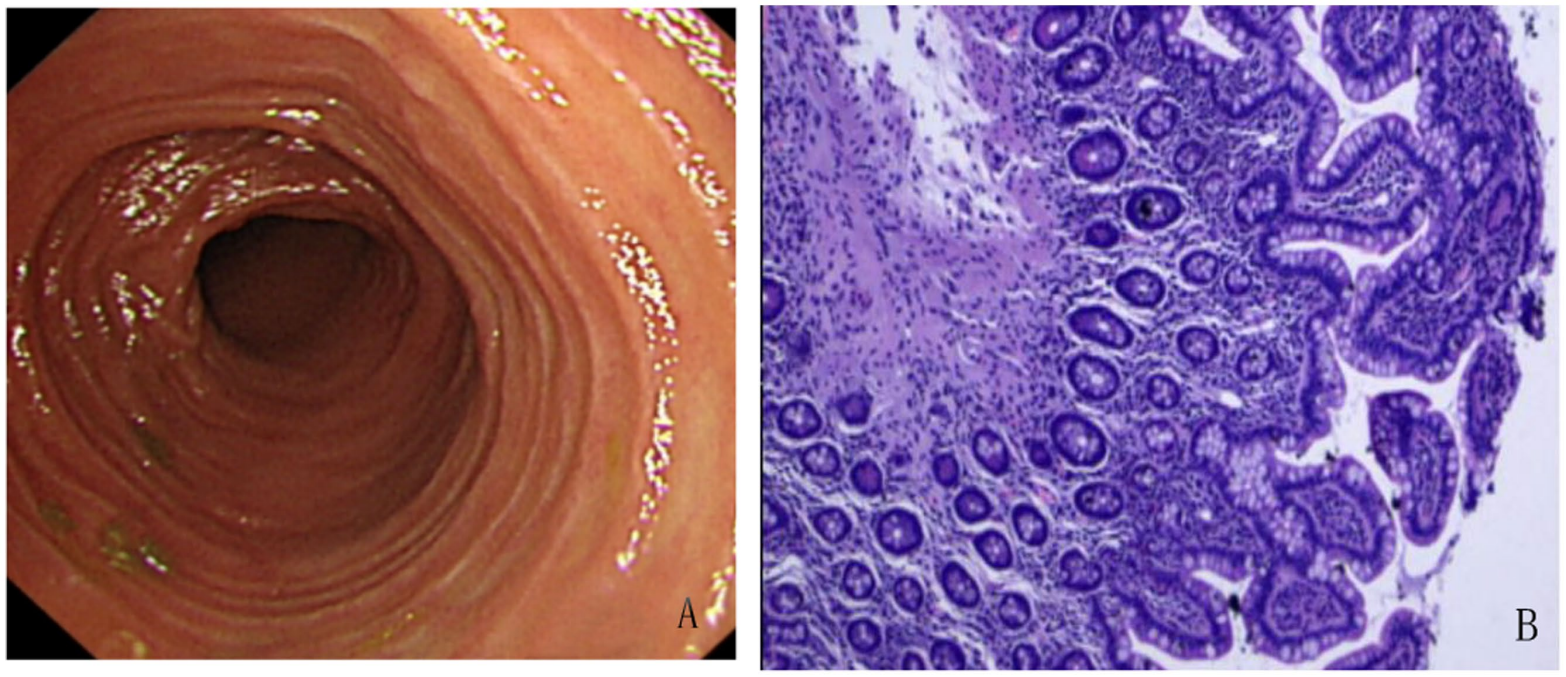
Small Bowel Enteroscopy and Histopathological Findings from Endoscopic Biopsy. **(A)** Enteroscopic examination revealed scattered lymphoid follicular hyperplasia in the terminal ileum. The surrounding mucosa appeared smooth, with well-preserved villous architecture and no evidence of ulceration, mass lesions, or abnormal vascularity. **(B)** Histological analysis of endoscopic biopsy specimens showed mild chronic inflammatory infiltration in the lamina propria with intact villous architecture. No significant plasma cell infiltration, granuloma, or vasculitis was identified. Lymphoid follicle formation was observed. There was no histological evidence of inflammatory bowel disease, intestinal tuberculosis, or Behçet’s disease.

Small bowel enteroscopy and endoscopic biopsy revealed no macroscopic evidence of tumor. Histological examination demonstrated mild chronic inflammatory infiltration of the small intestinal mucosa with preserved villous architecture, and no evidence of plasmacytosis, granuloma formation, or vasculitis. These findings were not indicative of GIST (Figure 1).

**Figure 2:**
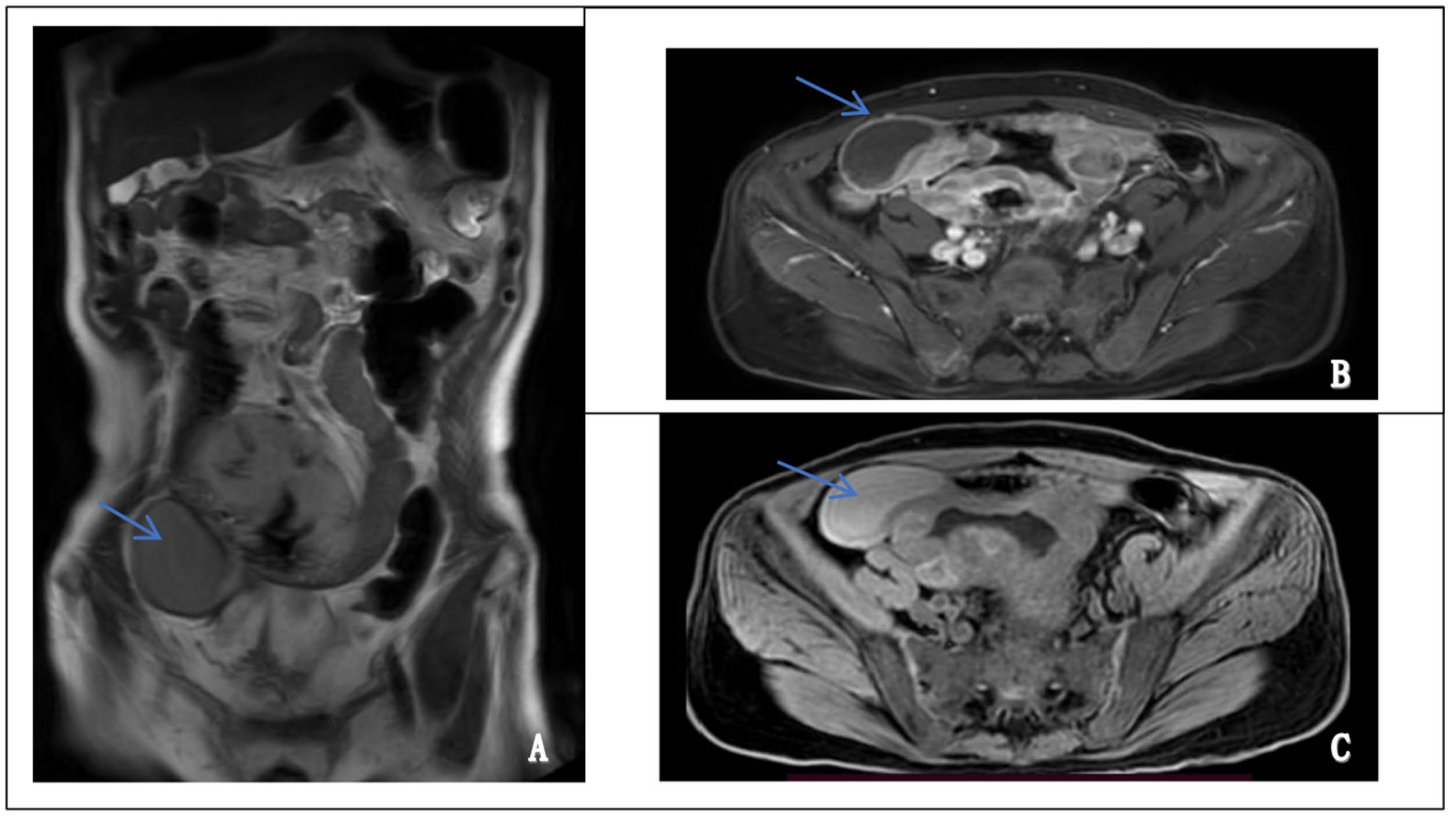
Magnetic resonance imaging (MRI) features of the abdominal mass. **(A)** Coronal T2-weighted image showing a well-defined, hyperintense mass in the lower abdomen (left panel, arrow). **(B)** Axial contrast-enhanced image demonstrating heterogeneous enhancement of the lesion, suggestive of a solid, vascularized tumor (upper right panel, arrow). **(C)** Axial T1-weighted image revealing a hypointense mass at the corresponding site (lower right panel, arrow).

Abdominal contrast-enhanced MRI performed on October 17, 2024, revealed an irregular, ring-enhancing mass located in the right lower quadrant. The lesion exhibited hyperintensity on T2-weighted images and hypointensity on T1-weighted images, with heterogeneous enhancement after contrast administration. Although intestinal lymphoma was initially suspected based on imaging features, there were no definitive signs of continuity between the mass and the intestinal lumen. A pelvic stromal tumor with central necrosis could not be excluded (Figure 2). These findings, in conjunction with inconclusive endoscopic biopsies, prompted surgical exploration for definitive diagnosis and treatment.

### Bone marrow biopsy

**Figure 3:**
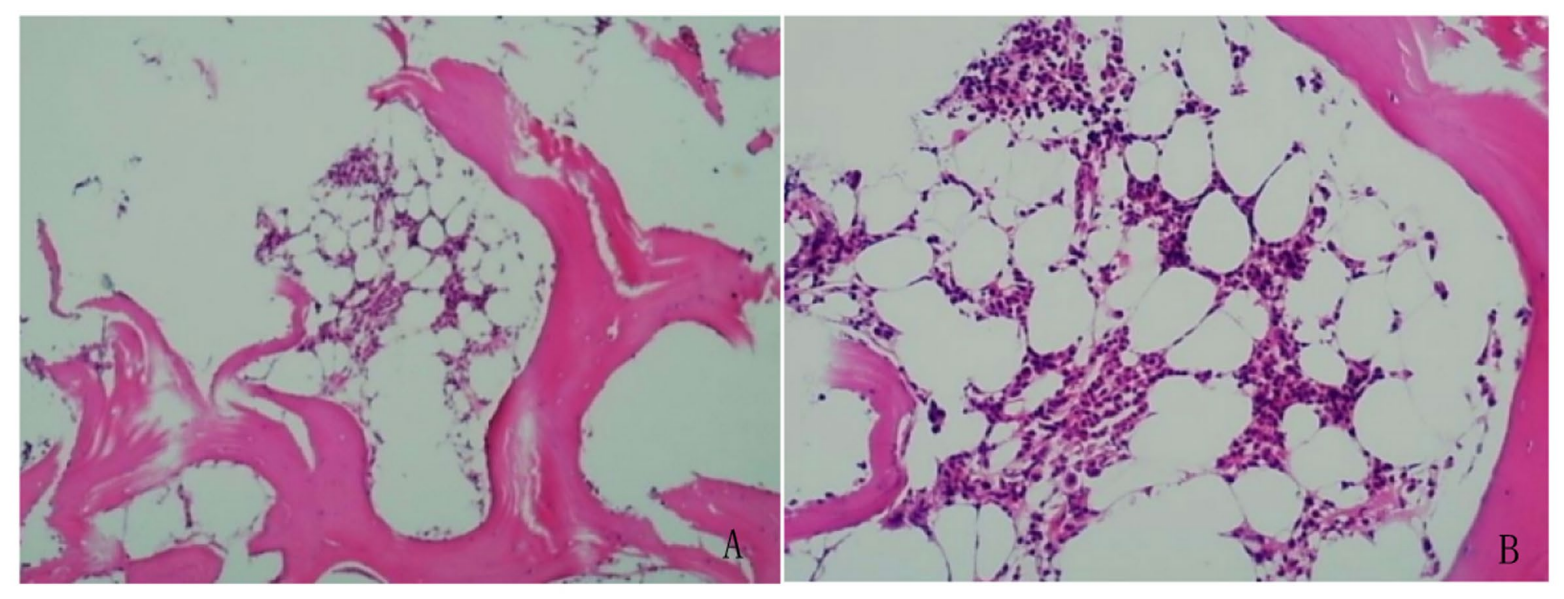
Bone marrow biopsy showed no evidence of hematologic malignancy. **(A)** Bone marrow cellularity was within normal limits, and megakaryocytes were adequate in number, exhibiting typical lobulated nuclei without dysplasia (left panel, H&E stain). **(B)** No abnormal increase or clustering of lymphocytes or plasma cells was observed, and no features suggestive of hematologic neoplasms were identified (right panel, H&E stain).

Microscopic examination of the bone marrow biopsy revealed mildly reduced hematopoietic activity, with nucleated cells occupying approximately 25% of the marrow space. The granulocytic series was sparse, predominantly consisting of mature forms. Similarly, the erythroid lineage appeared decreased. The number of megakaryocytes was within normal limits, with no evidence of abnormal clustering or dysplasia. No signs of bone marrow fibrosis were observed (Figure 3). Based on these findings, no features of hematologic malignancy were identified. The thrombocytosis was therefore considered to be reactive (secondary) in nature.

## Diagnostic thinking and treatment plan

### Differential diagnosis

Based on the clinical presentation and examination findings, the following diagnoses were considered in this case:

1. GIST: imaging suggested a solid tumor lesion, but endoscopic biopsy was inconclusive, requiring further examination.
2. Small intestinal lymphoma: imaging revealed abnormal signals in the small intestine, but biopsy showed no evidence of lymphoma.
3. Inflammatory bowel disease: although the patient exhibited abnormal bowel movements, there were no typical clinical features of inflammatory bowel disease, and pathological findings showed no characteristic changes.
4. Myeloproliferative disorders: bone marrow biopsy showed no characteristic features, indicating that the thrombocytosis was secondary in nature.

### Surgical decision

Due to the patient’s persistent abdominal pain and imaging suggestive of solid tumor lesions, but small colonoscopy biopsy failed to confirm the diagnosis, we made the final decision to perform partial resection of the small intestine and mesenteric lesion resection to alleviate the symptoms. Postoperatively, pathological biopsy and genetic testing were conducted to clarify the diagnosis.

## Surgical process and postoperative pathology

1.  Surgical procedure: partial resection of the small intestine and mesenteric lesions resection.
2.  Intraoperative findings

Intraoperative exploration found an isolated tumor (15x10x2cm) in the mesentery of the small intestine about 40 cm from the flexor ligament, with clear boundaries and no obvious plasma membrane invasion on the surface. We then performed a partial resection of the small intestine and removed the tumor completely. Intraoperative frozen section examination suggested a small intestinal stromal tumor.

3.  Postoperative pathology

**Figure 4:**
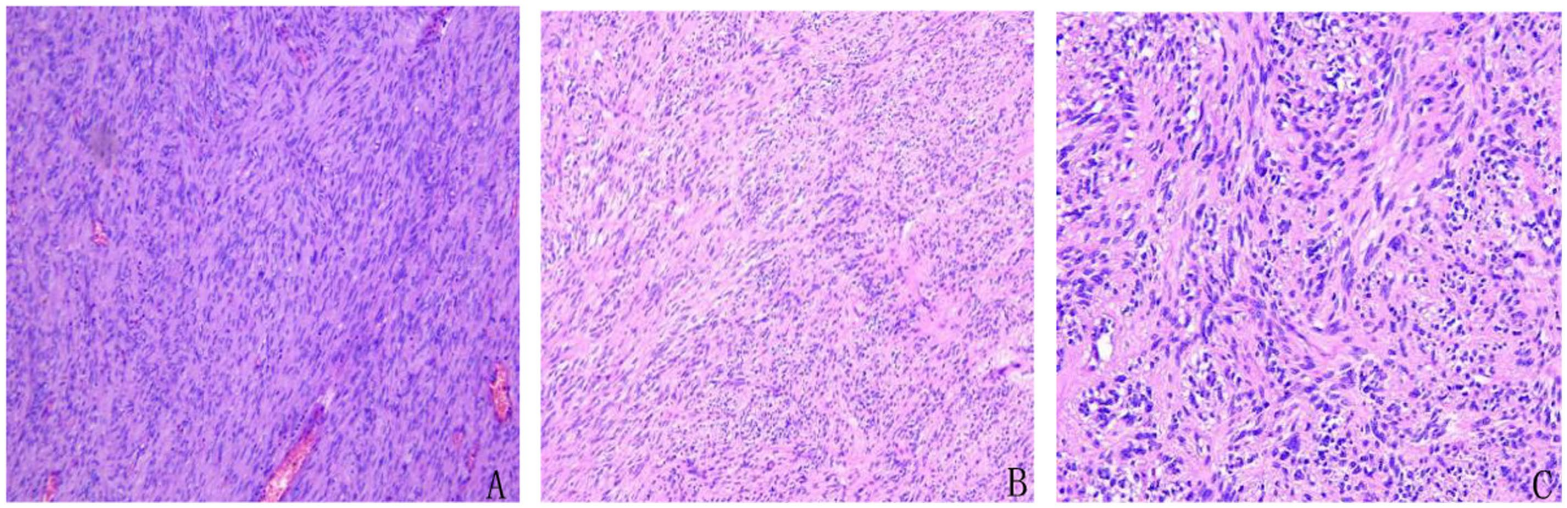
Histopathological features of the resected tumor showing classic morphology of gastrointestinal stromal tumor (GIST). Spindle-shaped tumor cells were arranged in fascicular and interlacing bundles. The cytoplasm was eosinophilic with indistinct cell borders. The nuclei were elongated or oval, and mitotic figures were readily observed, consistent with a high-risk GIST. **(A)** Hematoxylin and eosin (H&E), ×4. **(B)** H&E, ×10. **(C)** H&E, ×20.

Microscopic examination revealed spindle-shaped tumor cells arranged in fascicular and interwoven patterns. The cytoplasm was eosinophilic with indistinct cell borders, and the nuclei appeared spindle-shaped or ovoid. Mitotic figures were readily observed (Figure 4). Pathological diagnosis confirmed a GIST with high risk of recurrence. The tumor measured 15 × 10 × 2 cm, with a mitotic rate of >5 per 50 high-power fields (HPF) and areas of necrosis. There was no evidence of perineural or vascular invasion, and surgical margins were free of residual tumor. All four examined mesenteric lymph nodes were negative for metastasis.

**Figure 5:**
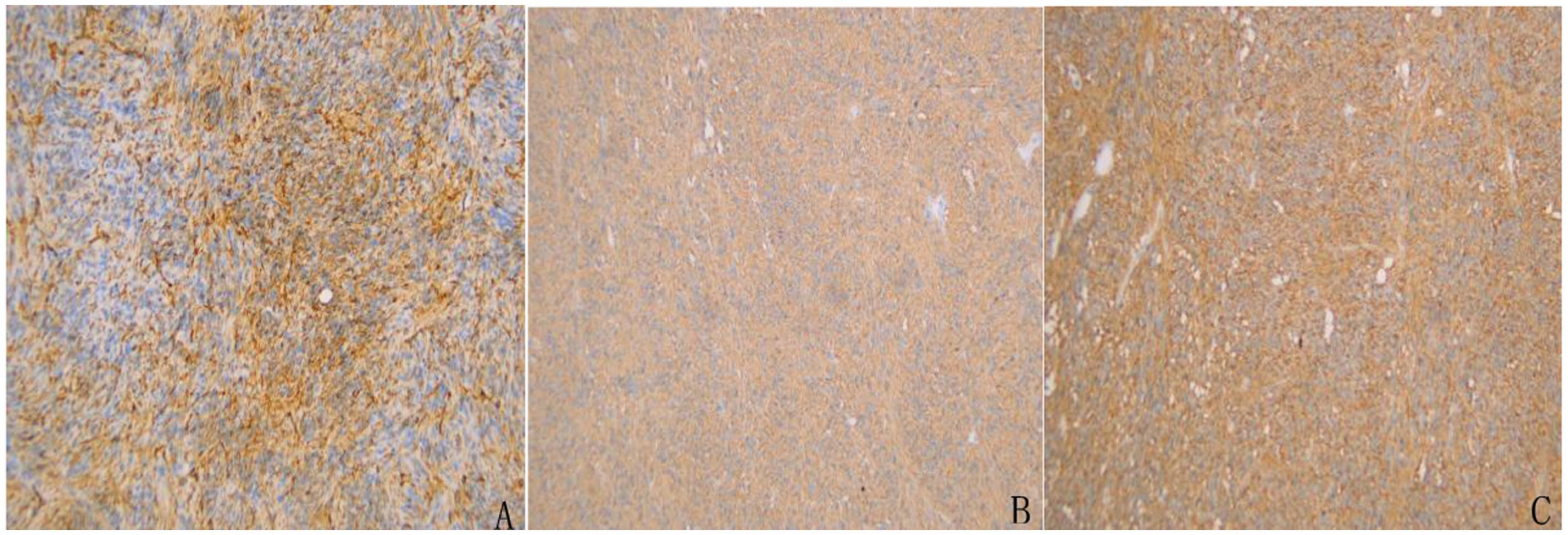
Immunohistochemical staining of the resected tumor specimen confirming the diagnosis of GIST. **(A)** CD34 staining showed partial positivity in tumor cells. **(B)** Strong and diffuse membranous positivity for CD117 (c-KIT) was observed. **(C)** DOG-1 staining was diffusely positive, further supporting the diagnosis of GIST.

Immunohistochemical staining demonstrated that the tumor cells were positive for CD117, DOG-1, CD34 (partially), Vimentin, and SMA, while negative for S-100, SOX-10, Desmin, CKp, and STAT6. TLE1 showed focal positivity, and SDHB was retained. The Ki-67 proliferation index was approximately 3%, suggesting low proliferative activity (Figure 5).

Genetic testing revealed a c-KIT gene mutation in exon 13, consistent with the diagnosis of GIST.

**Figure 6:**
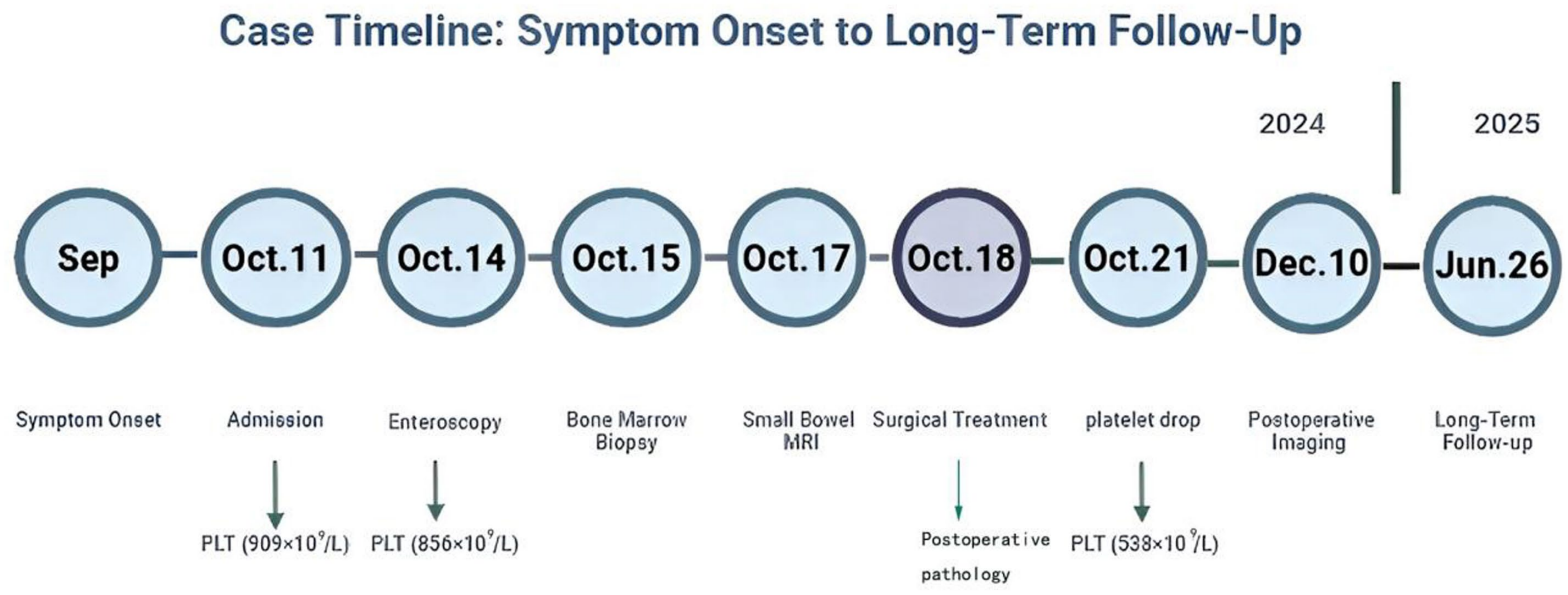
Timeline of the clinical course of the patient. The figure illustrates symptom onset, diagnostic workup, surgical treatment, and long-term follow-up. Platelet count values are annotated at key time points.

A detailed timeline of the patient’s symptom onset, diagnostic procedures, surgical treatment, and follow-up assessments is illustrated in Figure 6, providing a concise visual summary of the clinical course.

## Postoperative treatment and follow-up

The patient recovered well postoperatively without complications and resumed oral intake on the fifth postoperative day. According to the Fletcher risk classification, the case was classified as high-risk GIST; therefore, adjuvant therapy with oral imatinib mesylate (400 mg/day) was initiated to reduce the risk of recurrence.

During follow-up, multiple complete blood counts were performed, revealing a significant decline in platelet levels that returned to the normal range within 3 months after surgery (299 × 10^9^/L) (Table 1). Postoperative imaging on December 10, 2024 showed no signs of recurrence, suggesting that the thrombocytosis was closely associated with the tumor burden.

**Table 1:** Changes in blood counts.

Date	WBC (×10^9^**/**L)	HGB (g/L)	PLT (×10^9^/L)	RBC (×10^12^**/**L)
Oct 11, 2024	11.86	101	909	4.88
Oct 14, 2024	10.33	102	856	4.91
Oct 21, 2024	19.5	76	538	3.69
Nov 14, 2024	6.04	96	495	4.45
Nov 22, 2024	4.33	98	307	4.58
Dec 9, 2024	3.13	96	380	4.76
Dec 26, 2024	4.83	99	404	4.89
Jan 21, 2025	3.74	99	299	4.9
Feb 21, 2025	3.84	96	243	4.7
Mar 14, 2025	5.34	104	181	5.34
Apr 1, 2025	5.81	113	238	5.54
Apr 15, 2025	5.09	115	259	5.84
Jun 26, 2025	4.95	128	283	5.56

From a patient-reported perspective, symptoms such as abdominal pain and altered bowel habits resolved progressively, and the patient was able to resume daily activities within 8 weeks after surgery. No adverse or unanticipated events were observed during adjuvant therapy or follow-up. The patient reported satisfaction with the treatment outcome and a favorable overall quality of life.

## Discussion

### Correlation between GIST and secondary thrombocythemia

GIST is a rare gastrointestinal tumor originating from Cajal mesenchymal stromal cells, its pathogenesis mainly involves mutations in the c-KIT or PDGFRA genes (10–12). This case was diagnosed as c-KIT Exon 13 mutant GIST with significant thrombocytosis. Although GIST does not usually cause hematologic abnormalities, some patients may develop secondary thrombocytosis, the mechanisms may involve the following:

#### Tumor-related inflammation

Tumor tissues can induce a systemic inflammatory response by secreting pro-inflammatory cytokines such as interleukin-6 (IL-6) and interleukin-1β (IL-1β), which stimulate bone marrow activity and lead to increased platelet production. These pro-inflammatory factors not only promote the proliferation and differentiation of megakaryocytes, but also activate the synthesis of thrombopoietin, further accelerating platelet production (13, 14). IL-6 can stimulate the production of thrombopoietin in the liver and activate megakaryocytes in the bone marrow, thereby increasing platelet counts (15). IL-1β enhances this effect by promoting the interaction between tumor cells and immune cells in the tumor microenvironment (16). In this case, although preoperative IL-6 levels were unavailable, postoperative measurements showed a clear downward trend (130 pg./mL on postoperative day 1 to 12.7 pg./mL on day 8), paralleling the early postoperative decrease in platelet counts (Table 2). This temporal consistency supports the hypothesis that IL-6 may be involved in the thrombocytosis observed in GIST. However, it is important to note that current studies do not recommend routine perioperative measurement of IL-6 in GIST patients (17), and further studies are warranted to validate the clinical utility of IL-6 monitoring in this context. While several studies have identified IL-6 as a key mediator of cancer-related inflammation and thrombocytosis (18), its role in GIST-associated syndromes remains largely unexplored. Given the lack of consensus on IL-6 testing in routine GIST management, the findings in this case highlight a potential avenue for further investigation.

**Table 2:** Changes in IL-6 levels after surgery.

Date	Postoperative day	IL-6 (pg/mL)
Oct 19,2024	POD1	130.0
Oct 21,2024	POD3	78.7
Oct 24,2024	POD6	37.0
Oct 26,2024	POD8	12.7

POD, Postoperative Day.

#### Growth factor release

Tumor cells can secrete platelet-derived growth factor (PDGF), a potent growth-promoting factor that activates downstream signaling pathways by binding to the PDGF receptor, thereby stimulating the proliferation and differentiation of megakaryocytes. Within the tumor microenvironment, PDGF may collaborate with thrombopoietin to enhance platelet production (19). Previous studies have demonstrated that PDGF can promote thrombocytosis by stimulating bone marrow megakaryopoiesis. Moreover, PDGF plays a crucial role in the growth and metastasis of GIST, and may cooperate with c-KIT signaling to enhance tumor aggressiveness and promote oncogenic phenotypes (20, 21).

Although the present case exhibited marked thrombocytosis, PDGF levels were not assessed during the diagnostic and treatment process, reflecting the current lack of clinical emphasis on PDGF evaluation in GIST-related thrombocytosis. At present, there are no clinical studies recommending routine perioperative PDGF monitoring in GIST patients. Nevertheless, considering the potential involvement of PDGF in the development of thrombocytosis, further research is warranted to explore its utility as a serum biomarker for diagnosis, treatment monitoring, and prognosis in GIST.

#### Bone marrow compensatory hyperplasia

Patients with GIST often have chronic anemia, tumor invasion of gastrointestinal tissues may lead to prolonged gastrointestinal bleeding and malnutrition, ultimately resulting in chronic anemia. In this case, bone marrow cells compensate for the lack of hemoglobin by compensatory proliferation. Compensatory hyperplasia of the bone marrow not only enhances red blood cell production but may also stimulate megakaryocyte proliferation, leading to abnormal platelet elevation (22).

### Differential diagnosis of GIST with thrombocythemia

The patient exhibited significant thrombocytosis, which needs to be differentiated from primary and secondary thrombocythemia.

#### Myeloproliferative neoplasms

Bone marrow biopsy did not show JAK2, CALR or MPL mutations and there was no typical myelofibrosis or abnormal megakaryocyte hyperplasia, which excludes the possibility of myeloproliferative neoplasms.

#### Chronic inflammatory disease

The patient had abnormal bowel movements, but there was not obvious inflammatory response and the pathologic examination did not show chronic inflammatory disease.

#### Thrombocytosis associated with iron deficiency anemia

Although the patient had a slight decrease in hemoglobin, no significant abnormality was seen in the serum iron index, and the platelet level returned to normal after surgery, suggesting that thrombocytosis is associated with GIST.

### Clinical characterization and therapeutic strategy of Exon 13 mutant GIST

Mutations in the c-KIT gene are widespread in GIST, with Exon 11 mutations being the most common and Exon 13 mutations being less common, accounting for only about 1–2% of cases (2, 3). Several studies have shown that patients with Exon 13 mutations may exhibit a poorer therapeutic response to standard-dose imatinib (400 mg/d) compared to those with Exon 11 mutations, although some patients may still derive clinical benefit (23, 24). Therefore, therapeutic strategies for Exon 13 mutant GIST should take the mutational features into account, and individualized targeted therapy should be considered after surgery. Given that this case was a high-risk GIST, the standard dose of imatinib (400 mg/d) was still given as adjuvant therapy after surgery. For such patients, therapeutic efficacy should be closely monitored. If resistance or disease progression occurs during follow-up, increasing the dose to 800 mg/d or switching to a second-generation tyrosine kinase inhibitor, such as sunitinib, may be viable options (25).

Although KIT Exon 13 mutations have been reported in several retrospective studies and case series, they are typically described in the context of tumor location, response to therapy, or familial inheritance, and rarely in association with hematologic abnormalities (26). For instance, Yan et al. reported two rare Exon 13 variants, c.1926delA (p. K642*fs) and c.1936 T > G (p. Y646D), and examined their association with imatinib resistance through case reports and cell-based assays, but did not observe any hematologic abnormalities such as thrombocytosis (27). Ingley et al. (28) characterized a familial Exon 13 N655K mutation in a multigenerational GIST kindred and similarly found no evidence of platelet elevation. To date, no published studies have specifically linked Exon 13 mutant GIST with secondary thrombocytosis or other hematological manifestations. The present case may therefore represent the first reported instance of such an association.

This observation highlights a potential but previously undocumented clinical phenotype. The absence of similar findings in the literature suggests that thrombocytosis may be an underrecognized or rare manifestation in Exon 13 mutant GIST. Further accumulation of similar cases, ideally through multicenter registries or retrospective cohort studies, is essential to clarify whether this represents a reproducible clinical pattern or a coincidental finding. Such data would also support exploration of platelet levels as a potential prognostic biomarker in molecular subtypes of GIST.

### Prognosis and clinical significance of secondary thrombocytosis

Postoperative follow-up revealed that the patient’s platelet level returned to normal (299 × 10^9^/L) by 3 months postoperatively, demonstrating that it was a tumor-associated thrombocytosis rather than the result of a primary blood disorder. This phenomenon may be associated with postoperative regression of inflammation, decreased IL-6 levels and reduced tumor load. Dynamic changes in platelet levels may correlate with the course of GIST, therefore platelet levels may serve as potential biomarkers to guide clinical diagnosis and treatment (29, 30). Recovery of platelet levels in patients after surgery suggests a better short-term prognosis, but long-term follow-up remains essential as a means of assessing disease recurrence and the long-term efficacy of targeted therapies.

### Clinical implications

This case highlights a rare but potentially underrecognized clinical phenomenon: gastrointestinal stromal tumor (GIST) with paraneoplastic thrombocytosis. The patient presented with marked thrombocytosis and nonspecific abdominal symptoms, initially suspected to have intestinal lymphoma based on imaging. However, endoscopic biopsy was non-diagnostic, and bone marrow evaluation excluded hematologic malignancy. Definitive diagnosis of GIST was made only after surgical resection, emphasizing the need for comprehensive, multidisciplinary assessment in cases of unexplained thrombocytosis.

Importantly, postoperative normalization of platelet levels supported the tumor-related nature of thrombocytosis and aligned with clinical remission. This observation suggests that platelet trends may assist in monitoring disease course and therapeutic response in selected GIST cases. Furthermore, the presence of a c-KIT exon 13 mutation, a rare subtype with variable sensitivity to imatinib, underscores the importance of genetic profiling in guiding adjuvant therapy and surveillance strategies.

In clinical practice, persistent thrombocytosis without an identifiable hematologic cause should raise suspicion for solid tumors, including rare presentations of GIST. Integration of clinical, imaging, endoscopic, hematologic, and molecular data is essential for timely and accurate diagnosis, individualized treatment, and improved outcomes.

## Conclusion

In summary, GIST with secondary thrombocythemia is a rare clinical case, and it is even rarer to have a mutation in the c-KIT (Exon13) gene that exhibits significant platelet elevation. Therefore, we should follow up and report on such cases for more research in the future. When diagnosing and treating such patients, it is important to complete all relevant tests as thoroughly as possible, make careful decisions to minimize the risk of misdiagnosis, and develop a reasonable treatment plan. Although this study provides novel insights, it has certain limitations. It is based on a single case, which restricts the generalizability of the findings. While postoperative follow-up has been conducted, the long-term clinical course and durability of hematologic normalization remain to be fully clarified. Future clinicopathologic series or registry-based investigations are needed to determine whether thrombocytosis represents a consistent phenotype among GIST patients with KIT Exon 13 mutations or remains an incidental finding in rare cases.

## Data Availability

The original contributions presented in the study are included in the article/supplementary material, further inquiries can be directed to the corresponding author.
